# Nursing educators’ perception of disruptive behaviors in the professional work environment: A qualitative study

**DOI:** 10.30476/jamp.2021.89703.1383

**Published:** 2021-07

**Authors:** MARYAM TOLYAT, SEYYED ABOLFAZL VAGHARSEYYEDIN, MARYAM NAKHAEI

**Affiliations:** 1 Faculty of Nursing and Midwifery, Birjand University of Medical Sciences, Birjand, Iran

**Keywords:** Professionalism, Nursing, Bullying, Incivility, violence

## Abstract

**Introduction:**

Disruptive behaviors among nursing educators are a globally recognized problem. They have detrimental effects on nursing educators, the nursing profession, students and patients. This study aimed to explore nursing educators' experiences with disruptive behaviors in the professional work environment.

**Methods:**

The current study was conducted in 2019 and used a qualitative content analysis approach. Participants were selected purposely from nursing schools. Data was collected using semi-structured interviews with 20 nursing educators, and then analyzed according to the Graneheim and Lundman method.

**Results:**

Through analysis of the transcribed interviews, 4 categories and 10 subcategories were extracted. The categories include disrespectful interactions, inaccurate feedback on work performance, low acceptance in the clinical setting and perceived unfairness.

**Conclusion:**

Disruptive behaviors among nursing educators can affect professionalism as well as the quality of education provided by them. Therefore, considering factors that lead to disruptive behaviors in the professional work environment is necessary.

## Introduction

Negative workplace behaviors have been highlighted within the nursing profession in the past two decades ( 1). Studies have confirmed disruptive behaviors in various countries including the United States of America, Canada, Korea, Taiwan, Australia, Ireland, Italy and Singapore ( 2).

Various terms are used to describe this phenomenon, including abusive supervision, incivility, violence, aggression, bullying, petty tyranny and unfairness ( 3, 4). Workplace bullying has been defined as “behavior which is offensive, intimidating, intended to threaten, directed at a group of staff members, and occurring in relation to work” ( 5). Lower level negative workplace behavior is identified as incivility, and defined “as low-intensity deliberate behavior toward another with an intent to cause harm” ( 6).

Studies have reported varying incidence rates of disruptive behaviors among nurses as follows: 0.3% to 12% ( 7) as a daily occurrence; 14.7% ( 8) to 24.6% within the previous 6 months; 25.6% within the previous 12 months ( 9); and 57.1% experiencing sporadic exposure ( 7).

Disruptive behaviors have negative effects on nurses and cause issues such as: job dissatisfaction, diminished organizational commitment, emotional and physical problems, job burnout, depression, and turnover tendencies ( 4, 10). The negative impact of disruptive behaviors on the health of staff and patients is well documented and includes patient complications,, decreased staff and patient satisfaction and patient mortality ( 11).

In nursing education disruptive behaviors are also a serious issue. Nursing educators play vital roles in the nursing education ( 12). Disruptive behaviors can threaten the peace of educators in the educational and professional environment ( 13, 14). 

An accurate understanding of the types of disruptive behaviors would be helpful in developing strategies aimed to prevent disruptive behaviors in the academic setting.

Despite the importance of the negative effects of disruptive behaviors on employees and organizations, few studies have investigated disruptive behaviors in the academic setting. Thus, there is limited knowledge regarding disruptive behaviors among nursing educators.

Therefore, the present study was designed to explore nursing educators’ experiences with disruptive behaviors in the professional work environment. It is evident that disruptive behavior is a subjective concept, and two employees could display different interpretations of the same behavior ( 15). Thus, a qualitative study examining this concept can be helpful. Generally, qualitative studies are useful tools in providing holistic data that allows the researcher to understand the phenomenon as a whole ( 16).

## Methods

### Setting and participants

A conventional qualitative content analysis method was used to explore the experiences of nursing educators with disruptive behaviors in the professional work environment. Inclusion criteria comprised at least one-year of experience in academic teaching and a master’s degree or higher. Participants were selected purposely among nursing educators. The study settings were in the nursing schools. Participants signed written consent forms and were assured that they could leave the study at any time. After the 20th interview, the data was saturated, and no new information was added. Fourteen participants were female and six were male. 

### Data collection

Data was collected using semi-structured and in-depth interviews. Data collection was carried out between January 2019 and November 2020. The time and place of the interviews were selected by agreement with participants. Interviews were recorded by digital audio-recording. The interview was initiated with an open-ended primary question such as, “Please describe your experiences of a working day in the work environment?,” and “Please explain your experiences with any behavior in the workplace that has bothered you.” It was continued with more probing questions, such as, “Would you please explain more?” Interviews lasted approximately 60 to 90 minutes. All interviews were audio-recorded with the permission of the participants and transcribed verbatim by the researcher. The data collection process continued until saturation was reached and no new themes emerged.

### Data analysis

Data was analyzed using Graneheim and Lundman's content analysis method. The recorded interviews were listened to several times to obtain an in-depth and overall understanding. Then they were transcribed verbatim. The meaning units consisted of words, sentences, or parts of text, and were abstracted and labeled with a code. The various codes were compared based on differences and similarities in meaning and categorized based on this comparison. Similar codes were sorted into subcategories. Subsequently, similar subcategories were combined to create categories that were based on disruptive behaviors in the professional work environment. Data analysis was carried out using the MAXQDA10 software.

### Rigor

Lincoln and Guba’s criteria were used to ensure the validity and reliability of the data ( 17). To ensure credibility, the researcher had a long-term engagement (10 months), from January 2019 to November 2020, with the subject, data, and participants. Member check and expert check were also used after coding. After the data was analyzed, the emerging categories and subcategories were shared with the participants. For credibility, the transcribed interviews were returned to the participants after coding. It was confirmed that participants shared a common understanding about the research. The codes and categories were monitored and confirmed by a research team, which had sufficient experience in qualitative research. The researchers considered the maximum variation in sampling.

### Ethical considerations

The study was approved by the Ethics Committee of Birjand University of Medical Sciences, ethical code IR.bums.REC.1397.386. At the beginning of the interviews, the aim of the study was explained to the participants. Participants signed written consent forms that allowed their voices to be recorded during the interviews. They were assured that they could leave the study at any point and that their identities would be kept confidential by researchers.

## Results

Twenty nursing educators participated in the study. Fourteen participants were female and six were male. Thirteen participants had a master’s degree; the others had Ph.D. degrees. Seven nursing educators had managerial positions. The mean age of the nursing educators was 42.2±9/3 years, and their average work experience was 13.4±1.5 years.

Exploration of disruptive behaviors in the professional work environment of nursing educators resulted in 4 categories and 10 subcategories (Table 1).

**Table1:** Negative workplace behavior in the workplace of nursing educators

Category	Subcategory
Disrespectful interactions	Bullying
	Uncivil behaviors
	Violence in the clinical setting
Inaccurate feedback on work performance	Inappropriate performance appraisal
	A lack of appreciation
Lack of acceptance in the clinical setting	Ignoring the role of the educators in the care plan
	Rejection in the clinical setting
Perceived unfair	Discrimination in giving privileges
	Discrimination in allocation of type and number of credits
	Discrimination in payment

The experiences of nursing educators demonstrated that regardless of the type of disruptive behavior exhibited in varying degrees by managers, colleagues, students, patients and physicians, perceived disruptive behaviors occurred in the four forms discussed below.

### Disrespectful interactions

Most nursing educators stated that they had experienced disrespectful interactions in the professional work environment. Such disrespectful interactions came from managers, colleagues, students, patients and physicians. Nursing educators strongly complained about bullying, uncivil behaviors and violence in the clinical setting.

Bullying was mentioned as one of the main complaints of the participants. This subcategory included a range of behavior, such as excessive working expectations, uncoordinated assignment of the courses, exertion of mandatory educational program, and inadequate support.

 One female instructor stated: 

*“In the middle of the semester, they say, ‘Mr. /Ms. X cannot complete his/her internship. You should replace him/her.’ In the middle of the term, I had to go for an additional three-week internship in addition to my own internships.”*

Another female instructor said:

*“I have 16 weeks of internship during the semester. In addition, three days a week I have theory courses; moreover, practical units still remain.”*

Uncivil behaviors were another kind of disrespectful interactions, which predominantly originated from students. Uncivil behaviors and disregard for classroom norms disrupts the class and the learning process. These behaviors included arriving late to class or leaving early, mobile use, not paying attention in class, sleeping, being unprepared for class, absenteeism, challenging the educator’s knowledge, and talking to other students during class.

A male instructor stated: 

*“I even had a group that disrupted the class. They paid no attention to the lessons as if they were coming to class by force. They didn’t listen at all. Just this week, one of the boys was chewing gum.”*

Sometimes physicians behave violently verbally or non-verbally. Physical violence is manifested more as attacks by the patients’ companions or patients on the educator and students in the clinical setting.

As a male Associate Professor said:

*“We had an internship in the emergency . An accident patient was brought in. While we were doing the procedures for the patient, he died. The companion swore at us and started throwing whatever was in reach at us, shoes, anything. We were beaten well!”*

### Inaccurate feedback on work performance

Nursing educators perceived inaccurate feedback on work performance as the most common type of disruptive behavior, mostly perpetrated by managers. Nursing educators stated that they had experienced inappropriate performance appraisal in the professional work environment. Most educators stated that there is no appropriate criterion for assessing educators’ competence in the organization. Managers use quantitative criteria to evaluate educators, and they do not pay attention to the quality of the educators’ performance.

A male Associate Professor said:

*“In fact, I think quantity is much more important than the quality for the system. If I teach 30 credits a semester, I would be a good instructor. The one who teaches fewer courses would be the bad guy, no matter that the one who gets fewer courses may be teaching with higher quality. This won’t be seen.”*

Lack of appreciation was among the most common disruptive behaviors experienced by the participants. Despite the overly heavy workload and hard work by the educators, they believed managers were less appreciative of their efforts. Rarely did educators hear words of praise from their managers, and seldom was their hard work acknowledged by the managers. The ingratitude of managers had weakened the educators’ morale and degraded their efforts. One participating female instructor stated:

*“I’m trying so hard and I go home with swollen and painful legs. But the university never understands, they never thank me, they never do anything. The manager never realizes how a verbal appreciation could increase my motivation.”*

### Low acceptance in the clinical setting 

Nursing educators indicated that low acceptance in the clinical setting came from physicians and patients/their companions. Almost all the participants mentioned that being a nursing educator is not accepted in the clinical environment. Ignoring the role of the educators in the care plan and rejection in the clinical setting were expressed by nursing educators. 

 Nursing educators do not have a lot of organizational power in the clinical education environment, so they are subject to unreasonable expectations or demands of physicians and clinical staff.

An assistant professor stated:

*“Physicians disagree with the presence of nursing students in the ward. They don’t accept the nursing educator or a nursing Ph.D.; they turn their faces away and don’t talk to the educator. They frequently ask why there are so many nursing students in the ward.”*

Sometimes patients do not accept that students care for them because they do not have confidence in the competence of students.

One female instructor said:

*“Sometimes students want to perform a skill for the patient. The patient objects and says please do it yourself because they are still students.”*

### Perceived unfairness

Participants strongly complained about the unfair behavior of their managers. In fact, participants expected the managers to behave equally toward all nursing educators and to evaluate their performance justly. Participants believed that they have experienced discriminative behaviors by managers, including discrimination in giving privileges, allocation of type and number of credits, and perceived unfairness in pay.

A male instructor stated:

*“Some educators do not go for internships. The workload is a burden on the shoulders of some certain colleagues; this pressure is not shared by everyone. The rules do not apply to everyone equally.”*

Perceived unfairness in pay was another unfair behavior about which some participants complained. They believed that the amount of pay varies greatly between academic degrees despite the same job responsibilities. They considered the unfairness of decision-making procedures, particularly the payroll system, as the primary contributor to this situation.

A female instructor said:

*“There is a huge gap between the salaries of an instructor, assistant professor, associate professor and professor/full professor. I think the payroll system needs to be revised.”*

## Discussion

The purpose of this study was to describe nursing educators’ experiences with disruptive behaviors in the professional work environment. This study found that nursing educators perceived disruptive behaviors on the part of managers, students, patients, patients’ relatives, or physicians in the form of disrespectful interactions, inaccurate feedback on work performance, low acceptance in the clinical setting and perceived unfairness. Various studies have referred to disruptive behaviors ( 18- 20). 

Various studies have defined disruptive behavior or toxic behavior as bullying, threats, unfairness and discrimination, aggression, narcissism, unethical behaviors, unrealistic expectations, aggressiveness, intimidation and making incorrect appraisals regarding their work performance ( 21, 22).

In this study, one of the categories extracted was disrespectful interactions, which included bullying, uncivil behaviors, and violence in the clinical setting.

The behaviors experienced by nursing educators in the form of bullying included excessive working expectations, uncoordinated assignment of courses, exertion of mandatory educational program, and inadequate support. Various studies have confirmed the existence of workplace bullying in nursing and other work environments. The dimensions identified in other studies for bullying include insult and humiliation, hatred, threatening, boring and unnatural workloads ( 23- 25). Richards (2016) noted that high workload is an issue that impacts retention of nursing educators. Various studies described the workload of nursing educators as overwhelming and exhausting with low levels of satisfaction ( 26).

 It seems that bullying experienced by nursing educators differs slightly from what happenings in other workplaces. Notably, bullying by a manager is not always visible and may be a passive act, such as a failure to support the employee ( 27, 28). Sometimes, bullying in higher education can be very subtle and clever; the perpetrators may even seem compassionate so that their victims are not aware of what is happening ( 29). In addition, national culture may play a crucial role in the perception of bullying by educators.

In Iran, bullying occurs mainly in top-down processes, in which the target is usually in a lower position than the perpetrator ( 30). Moreover, the lack of funds precludes the recruitment of a sufficient number of nursing educators. Therefore, excessive work expectations from educators by managers are due to the lack of educators in the workplace.

Uncivil behaviors identified included arriving late to class or leaving early, mobile use, not paying attention in class, sleeping, being unprepared for class, absenteeism, challenging the educator’s knowledge, and talking to other students during class which support previous findings ( 13, 31). Minor differences between the current study and other studies may be due to cultural differences between the studied societies ( 32).

Nursing educators participating in the current study reported physical and verbal violence from patients/their companions in clinical settings. As nursing educators are part of the care system and spend most of their time working with students in the clinical setting, they also experience some of the violence in the care system, despite slight differences. Many studies on violence in nursing have indicated that nurses experience violence in the workplace ( 33, 34).

Inaccurate feedback on work performance was the most common form of disruptive behavior perceived by nursing educators. There are few studies examining inappropriate performance appraisal among employees working in health care organizations ( 35, 36). In the current study nursing educators were dissatisfied with the performance appraisal process and less motivated in their work. They also experienced a lack of appreciation for their activities. Like other professionals, nursing educators find it justifiable to be appreciated for what they do. Appreciation creates a sense of worth for the employee in the workplace, ultimately leading to enhanced efficacy of the educators and improved status of the institution ( 37).

Low acceptance in the clinical setting was another finding of this study. Behaviors related to low acceptance in the clinical setting included ignoring the role of educators in the care plan and rejection in the clinical setting by patients and physicians. Nursing educators pointed out that ignoring the role of the nursing educator in the care plan is a common phenomenon because there is a physician-dominated atmosphere in the clinical setting and nursing educators do not have enough organizational power in the clinical education environment. As a result, they are subject to unreasonable expectations or demands of physicians and clinical staff. Moreover, nursing educators employ a cautious set of manners with staff and physicians to prevent conflict in the clinical setting.

Perceived unfairness in the workplace was one of the forms of disruptive behaviors. Unequal work atmosphere and discrimination were also frequently mentioned as being perceived disruptive behaviors among nursing educators. They stated that managers are mostly responsible for causing such types of atmosphere. In Keasly’s study (2010), 38% of employees confirmed the manager’s unfair behavior in higher education ( 38). Previous studies have reported unfair behaviors as displayed in performance evaluation, use of reprimand, distribution of educational resources, anger with employees, mission assignment and transfer of employees, working hours, forced planning and working hours, administrative duties, and pay ( 39, 40).

This study revealed perceptions of discrimination in the assignment of the type and number of courses between nursing faculties. Due to the difficulty of a clinical education ( 41), educators are less inclined to teach clinical courses. Hence, such courses are usually assigned to educators with less work experience and lower educational qualifications, which causes dissatisfaction among nursing educators. 

Disruptive behaviors, even at a low level, can leave a substantial effect on the professionalism among nurse educators and nursing students. Professionalism has become key issues in health care systems ( 42). One’s values are shaped by one’s experiences, affect one’s behavior and interactions with others, and are manifested in professional behavior ( 43) . In other words, nursing educators play a key role in preparing students for graduate nursing practice ( 44). New nurses are expected to display behaviors of professionalism; thus, nursing schools should help students to internalize these behaviors. Nurse educators carry a responsibility to shape future nurses’ growth towards professionalism ( 43). Therefore, attention to professionalization is necessary ( 44). It is essential to recognize such behaviors and their effect on the professionalism among nurse educators in the professional work environment. 

The current researchers suggest that this study be conducted in other nursing schools. It is further recommended that interventional studies be conducted with the aim of managing annoying behaviors in nursing educators’ work environments. The findings of this study should be considered by faculty and university managers. 

One of the limitations of the present study was the educators’ reluctance to share their experiences of disruptive behaviors. Sometimes, the participants even refused to speak about some abusive experiences. In such circumstances, the researcher attempted to ask general and indirect questions to discover the educators’ experiences. Moreover, the use of the qualitative approach limits the generalizability of the findings.

## Conclusion

In the current study, the perspectives of Iranian nursing educators' regarding disruptive behaviors were gathered. Nursing educators experienced threats to human dignity in the form of disrespectful interactions, inaccurate feedback on work performance, low acceptance in the clinical setting and perceived unfairness in the professional work environment. Disruptive behaviors can affect the educational, organizational and professional environment, leading to a failure in achieving the goals of professional organization. The findings of the present study can help to better understand disruptive behaviors in the professional work environment from the perspective of nursing educators in Iranian culture. Consequently, managers should design strategies and policies to minimize disruptive behaviors in nursing schools. Also, managers of nursing schools should provide a supportive environment in which educators feel that they are valued and can work to achieve their potential. These findings may differ from the perceptions of nursing educators in other countries.

## Ethical approval

The study was approved by the Ethics Committee of Birjand University of Medical Sciences, ethical code IR.bums.REC.1397.386.
